# Antibiotic Prescription Audits Among Pediatric Outpatients With Acute Ailments in a Secondary Care Hospital During the COVID-19 Omicron Wave in Northern India

**DOI:** 10.7759/cureus.32017

**Published:** 2022-11-29

**Authors:** Baani Sodhi, Saurav Basu

**Affiliations:** 1 Indian Institute of Public Health-Delhi (IIPH-D), Public Health Foundation of India, New Delhi, IND

**Keywords:** health public, covid-19, prescription audit, antibiotics therapy, antibiotic resistance (abr)

## Abstract

Background

Antibiotics, as defined by the World Health Organization (WHO), are pharmaceuticals used to treat bacterial infections. There is growing recognition that inappropriate antibiotic prescription in children is linked to increasing rates of severe adverse drug events and higher medical expenditures. There are a few prescriptions audit studies from smaller cities in Northern India, especially those conducted during the COVID-19 pandemic when the unregulated private sector accounted for 90% of antibiotic sales and 75% of healthcare requirements. The study objectives were to determine the rate of outpatient antibiotic prescription and adherence to WHO drug indicators in prescriptions to pediatric outpatients in private healthcare facilities in India.

Methodology

This cross-sectional survey was conducted over three months (January to March 2022) in the outpatient setting of a private pediatric hospital in Kanpur, a city having a population of nearly three million population located in the state of Uttar Pradesh in India. Prescriptions of children aged <10 years with a history of onset of complaint <14 days were included in this audit. Prescriptions were numbered; data were collected using a specially designed semistructured, pretested prescription audit checklist; and the recommended WHO indicators were also calculated. Data were entered using CSPro (U.S. Census Bureau, Washington, DC, USA) and analyzed using STATA 15 (StataCorp LLC, College Station, TX, USA).

Results

This study observed an antibiotic prescription rate of 65.75%, which was higher than the WHO-recommended value, which might indicate indiscriminate usage of antibiotics in the setting. Out of the 144 antibiotic medications prescribed, none were generic and all the antibiotics were prescribed presumptively. The most commonly prescribed medicines were cefpodoxime, azithromycin, and ofloxacin, which were primarily used to treat cough and stomach infections.

Conclusions

This antibiotic audit conducted in a private hospital outpatient setting in a city in Northern India during the Omicron wave of the COVID-19 pandemic found nongeneric, predominantly oral, presumptive antibiotic prescriptions in nearly two out of three young pediatric patients. Improvement in prescribing practices through regulation, monitoring, and antibiotic stewardship in low-resource settings is urgently warranted to curb the impending global pandemic of antimicrobial resistance.

## Introduction

Antibiotics, as defined by the World Health Organization (WHO), are pharmaceuticals used to treat bacterial infections [1]. Antibiotics are routinely prescribed to children in outpatient settings to treat common pediatric infections, especially respiratory tract, ear, and diarrheal infections, to reduce disease severity and improve patient outcomes [2-4]. It is estimated that each year, over 65 million doses of antibiotics are prescribed to children in outpatient settings, with a significant proportion of these courses being unwarranted and attributed to antibiotic overuse, particularly in the developing world with a lack of effective oversight [5].

In India, within the private healthcare sector, it is estimated that over 519 million antibiotic prescriptions are written annually, with a high rate of systemic antibiotic prescriptions, especially in the age group 0-4 years [6]. The per-capita antibiotic consumption in the Indian retail sector was observed to increase from 13.1 defined daily doses per 1,000 inhabitants per day (DID) in 2008 to 16.0 DID in 2012 (a 22% increase), along with increased consumption of new classes of antibiotics [7].

Irrational prescription of antibiotics is strongly linked with triggering accelerated antibiotic resistance, a phenomenon through which microorganisms induce changes in themselves that render them protection against prior antibiotic susceptibility [1,8-10]. Furthermore, there is growing recognition that inappropriate antibiotic prescription in children is linked to increasing rates of severe adverse drug events and higher medical expenditures [11]. Moreover, prescribing errors contribute to a further increase in patient morbidity and mortality [12-14]. Misuse of medications, especially antibiotics, is frequently an outcome of ineffective regulation and lack of sensitization and education of stakeholders, including prescribers, consumers, and dispensers, suggestive of the need for diverse intervention measures [15,16].

During the COVID-19 pandemic, globally, there has been a substantial increase in antibiotic prescriptions for both prophylaxis and active management of COVID-19 despite the proven lack of efficacy of antibiotic formulations against a viral disease [17]. In India, the unregulated private sector accounted for 90% of antibiotics sales and 75% of healthcare services in this period [18,19].

The WHO recommends the assessment of drug use practices as part of an audit process in healthcare systems through the measurement of standardized indicators to curb drug misuse and improve the quality of healthcare [20]. The prescription audit is a systematic critical analysis to assess the quality of medical care. The rational use of drugs is based on the rule of right: right drug, right patient, right dose, and right cost [21].

There are a few prescriptions audit studies from smaller cities in Northern India, from private care facilities, especially those conducted during the COVID-19 pandemic. The objectives of this study were to determine the rate of outpatient antibiotic prescription and adherence to WHO drug indicators in prescriptions to pediatric outpatients in private healthcare facilities in India.

## Materials and methods

Design and setting

This prescription audit was part of a larger cross-sectional survey conducted in the outpatient setting of a private pediatric hospital in Kanpur, a city having a population of nearly three million located in the state of Uttar Pradesh in India. The facility was catering to patients across all economic strata, and most respondents had no insurance coverage. The average total patient count per day was 20. The study was conducted during the Omicron wave of the COVID-19 pandemic in India from January to March 2022.

Study participants

Prescriptions of children aged <10 years with a history of onset of complaint <14 days were included in this audit. Exit interviews were also conducted with the parents or caregivers of the children. Children reporting complaints relating to a chronic illness or those referred from any other healthcare facility were excluded. None of the patients reported a diagnosis of active COVID-19 infection although some had submitted samples to the diagnostic laboratories and were awaiting their test results.

A total of 219 face-to-face interviews were conducted, with a response rate of 100%, with nearly seven prescriptions assessed per day by consecutive sampling. The high response rate was due to the recommendation by the treating physician. The WHO recommends a sample size of at least 100 outpatient encounters in an individual health facility for assessing patient care indicators [22]. 

Study outcomes and operational definitions

The following indicators were calculated using the formulas recommended by the WHO [22]:

· Percentage of medicines prescribed by generic name: This was calculated as the ratio of the number of drugs prescribed by generic name to the total number of drugs prescribed, multiplied by 100.

· Percentage of antibiotics per prescription (Antibiotics were classified based on the WHO model list for antibiotic classification.): This was calculated as the ratio of the number of patient encounters in which an antibiotic would be prescribed to the total number of encounters surveyed, multiplied by 100.

· Percentage of injections per prescription (Vaccinations were excluded from this list.): This was calculated as the ratio of the number of patient encounters in which an injection would be involved to the total number of encounters surveyed, multiplied by 100.

· Percentage of fixed-dose combinations (FDCs) prescribed: This was calculated as the ratio of the number of patient encounters in which FDCs were prescribed to the total number of encounters, multiplied by 100.

· The quality of prescriptions was evaluated by assessing the prescription legibility. Prescriptions were graded as Grade 1 (legible with ease), Grade 2 (legible with difficulty), and Grade 3 (illegible).

Methodology

Data were collected every day from prescriptions of pediatric patients after obtaining parental consent. Prescriptions were numbered, and data were collected using a specially designed semistructured, pretested *prescription audit checklist* that was adapted from the WHO and the United States Agency for International Development-funded antimicrobial stewardship program toolkits and guidelines for low- and middle-income countries [22,23]. Information extracted included patient details, diagnosis, sex of the patient, disease for which antibiotics were prescribed, type of antibiotics prescribed and its dose, dosage form, route, sensitivity analysis, and legibility of prescriptions.

Statistical analysis

Data were entered and analyzed using STATA 15 (StataCorp LLC, College Station, TX, USA). Mean, percentages and frequency, median, and ranges were used to present a descriptive analysis of the variables.

Ethical considerations

The study was approved by the Institutional ethics committee Indian Institute of Public Health, Delhi (Approval No. IIPHD_IEC_S_01_2021 ). Written and informed consent was obtained from all the study participants.

## Results

A total of 219 pediatric outpatient prescriptions were assessed in this study. The age of the pediatric patients in the study was mean = 3.46 (SD 2.79) years (ranging from 1 month to 9.5 years), and of the 219 patients, 139 (63.47%) were males and 80 (36.53%) were females. Clinical history was mentioned only in 66 (30.14%) of the prescriptions and blood or urine culture was not recommended in any of the cases. Antibiotics were prescribed in 144 (65.75%) cases, with 64 (44.4%) prescribed for the common cold. The majority (97.22%) of the antibiotics were for an oral mode of administration (Table 1).

**Table 1:** Characteristics of the prescriptions audited.

Variable	*n* (%) (*N* = 219)
Complete name of the patient present	
Yes	62 (28.31)
No	157 (71.69)
Age of child in years, mean (SD)	3.46 (2.79)
Weight of child in kg, mean (SD)	14.02 (8.02)
Gender of the child	
Male	139 (63.47)
Female	80 (36.53)
Clinical history present	
Yes	66 (30.14)
No	153 (69.86)
Clearly mentioned diagnosis	
Yes	69 (31.51)
No	150 (68.49)
Antibiotics prescribed	
Yes	144 (65.75)
No	75 (34.25)
Disease for which antibiotics were prescribed (n = 144)	
Diarrhea and vomiting	52 (36.11)
Common cold	64 (44.44)
Stomach pain	7 (4.86)
Others	21 (14.58)
Type of antibiotic prescribed (n = 144)	
FDC	21 (14.58)
Individual	123 (85.42)
Both	0 (0)
Duration of antibiotics, mean (SD) (n = 144)	3.33 (1.00)
Number of antibiotic injections	0 (0)
Sensitivity analysis performed before antibiotic prescription	0 (0)
Total dosage of antibiotics (mg; n = 144), median (IQR)	600 (750)
Primary mode of antibiotic medicine (n = 144)	
Local	4 (2.78)
Oral	140 (97.22)
Total days of treatment in days, mean (SD) n = 144)	5.60 (4.17)

The most commonly prescribed antibiotics by the treating physician were cefpodoxime, azithromycin, and ofloxacin, which were primarily used to treat cough and stomach infections.

WHO indicators were calculated from the audited prescriptions based on standard formulas. A total of 21 (14.58%) prescriptions had prescribed medicines as FDCs, and none of the prescriptions included generic medicines. There were 185 (84.47%) prescriptions having a physician’s grade 1 handwriting, i.e., they were legible with difficulty (Table 2).

**Table 2:** WHO indicators. FDC, fixed-dose combination; WHO, World Health Organization

Variable	n (%) (N = 144)
Percentage of antibiotics per prescription	144 (65.75)
Percentage of generic medicines	0 (0)
Percentage of injections per prescription	0 (0)
Percentage of FDCs	21 (14.58)
Doctor’s handwriting grade (n = 219)	
Grade 1 (Legible with ease)	0 (0)
Grade 2 (Legible with difficulty)	185 (84.47)
Grade 3 (Illegible)	34 (15.53)

The antibiotic prescription rate of the study was higher than the WHO-recommended values although injectables were not prescribed at all (Table 3).

**Table 3:** Comparison of WHO drug use prescribing indicators. WHO, World Health Organization

Prescribing indicators	Optimal values	Values obtained in the study
Medicines prescribed by generic name (%)	100	0
Antibiotics per prescription (%)	<30	65.75
Injections per prescription (%)	<20	0

## Discussion

A medical audit is the examination of the medical and healthcare systems toward eliciting improvement in the standard of healthcare services provided to patients. The prescribing practices are crucial as only rational prescriptions can ensure therapeutic efficacy and safety [24].

In this study, nearly two in three prescriptions included antibiotics. Secondary data analysis of a nationally representative pharmaceutical sales database from India found the rate of antibiotic prescription in children aged 0 to 4 years was 636 prescriptions per 1,000 persons, which is similar to our study [7]. In this study, the high rate of antibiotic prescription may also be due to the then ongoing Omicron wave of the COVID-19 pandemic in India and other low- and middle-income countries (LMICs) owing to raised levels of social anxiety and lack of adherence to evidence-based medicine by prescribers apart from increased over-the-counter sales [17,19,25].

Illegible prescriptions are a major cause of medication errors and avoidable cause of harm to patients [6,12]. Previous Indian studies show a pattern of significant variation in the proportion of illegible prescriptions. A prescription audit study from the state of Kerala reported only 3.4% (122/3,557) prescriptions as illegible [17]. In comparison, this study reported the proportion of illegible prescriptions as nearly 4.5 times higher, a finding similar to another study from the outpatient setting of a teaching hospital in Southern India [26].

In LMICs, antibacterial medicines are commonly administered to children in outpatient settings for the treatment of common pediatric illnesses (colds, upper respiratory tract infections, and bronchitis) that usually do not warrant antibiotic treatment due to viral etiology [2-4,26,27]. In agreement with this, our study also found high rates of presumptive antibiotic prescribing patterns for similar symptomatic profiles.

No injectable medications were prescribed in our study setting, which is much lower than previous studies [24,27] as our study focused on only children aged <10 years within outpatient settings, whereas other studies focused on adults.

No generic antibiotic medication prescriptions were observed in this audit. The WHO recommended individual countries develop their own locally appropriate national medicines policy that primarily focused on improved access to the high-quality and rational use of generic medicines [28]. Even though there has been a recent thrust toward promoting high-quality and low-cost generic medications in India through major government initiatives [29], patient and provider perceived concerns about their safety and efficacy compared with branded drugs diminish their acceptability [20,30].

This study's strengths include data collection from a private healthcare setting of a smaller city in Northern India and study sites that are usually neglected by previous such research in India, which were conducted in government health facilities or larger private hospitals with multiple providers. However, these smaller healthcare facilities in urban and suburban areas of the country provide cumulative health access to millions of people and are potential drivers of antibiotic resistance through presumptive antibiotic overuse by practitioners.

Additional strengths of this study are a response rate of 100%, which ensured there was no missing data. The use of validated forms ensured that the data collected are standardized and comparable with previous studies. As the study site hospital catered to patients from all economic backgrounds, the study population was more representative of the local community.

Study limitations are the lack of external validity as the study was conducted in only a single private outpatient setting in Kanpur city and focused only on pediatric (aged <10 years) prescriptions and had a relatively smaller sample size for assessing prescribing indicators.

## Conclusions

In conclusion, this antibiotic audit conducted in a private hospital outpatient setting in a city in Northern India during the Omicron wave of the COVID-19 pandemic found nongeneric, predominantly oral, presumptive antibiotic prescriptions in nearly two out of three young pediatric patients. Improvement in prescribing practices through regulation, monitoring, and antibiotic stewardship in low-resource settings is urgently warranted to curb the impending global pandemic of antimicrobial resistance. 
